# The role of RNA m6A methylation in lipid metabolism

**DOI:** 10.3389/fendo.2022.866116

**Published:** 2022-09-08

**Authors:** Yuting Wang, Yujie Wang, Jiarui Gu, Tianhong Su, Xiaosong Gu, Yu Feng

**Affiliations:** ^1^ Department of Endocrinology, The Second Affiliated Hospital of Soochow University, Suzhou, China; ^2^ Department of Orthopaedics, Dushu Lake Hospital Affiliated to Soochow University, Suzhou, China; ^3^ Department of Cardiology, the Second Affiliated Hospital of Soochow University, Suzhou, China

**Keywords:** METTL3, obesity, FTO (fat mass and obesity-associated) gene, M6A, lipid

## Abstract

The m6A methylation is the most numerous modification of mRNA in mammals, coordinated by RNA m6A methyltransferases, RNA m6A demethylases, and RNA m6A binding proteins. They change the RNA m6A methylation level in their specific manner. RNA m6A modification has a significant impact on lipid metabolic regulation. The “writer” METTL3/METTL14 and the “eraser” FTO can promote the accumulation of lipids in various cells by affecting the decomposition and synthesis of lipids. The “reader” YTHDF recognizes m6A methylation sites of RNA and regulates the target genes’ translation. Due to this function that regulates lipid metabolism, RNA m6A methylation plays a pivotal role in metabolic diseases and makes it a great potential target for therapy.

## Introduction

Lipid metabolism exerts a profound impact on the maintenance of human physiology and health status. Adipose tissue is an important site for lipid storage, and energy homeostasis (1, 2). It is important to understand the mechanisms involved in adipose tissue development (3). Adipogenesis of the white and brown adipocytes is regulated by several endocrine hormones (1, 3). Fat mass and obesity-associated protein (FTO) pro-obesity rs1421085 T-to-C single-nucleotide polymorphism (SNP) shifts differentiation programming towards white adipocytes in subcutaneous fat (4). Meanwhile in community, unhealthy lifestyles such as nutrient surplus and unhealthy eating patterns (5) act as the main reason for the high incidence of lipid metabolism disorder. Furthermore, types of diseases caused by abnormal lipid metabolisms like diabetes (6), hyperlipidaemia (7), cardiovascular disease (8, 9), and non-alcoholic fatty liver disease (NAFLD) (10) are becoming more and more pervasive all over the world. Therefore, there is a great desire to deepen the understanding of the regulation of lipid metabolism.

In mammals, the m6A methylation is the most numerous modification of mRNA and accounts for more than sixty percent of all RNA modifications (11, 12). The RNA m6A modification is a kind of methylation modification positioned at the nitrogen atom in the sixth position of adenosine (13). The process of RNA m6A methylation is dynamically and reversibly coordinated by m6A demethylases, m6A methyltransferases, and m6A binding proteins, which are also referred to as “Writer”, “Eraser”, and “Reader”, respectively (14). The writers methyltransferase-like 3 (METTL3), methyltransferase-like 14 (METTL14), and Wilms’ tumor 1-associated protein (WTAP) have m6A methylation activity to catalyze m6A modification (15). Demethylases are predominantly made out of ALKB homolog 5 (ALKBH5) and FTO (16), catalyzing the demethylation process (17). Furthermore, m6A binding proteins are found principally in the YT521-B homology (YTH) family (18), which have the potency to recognize and specifically bind to m6A-modified transcripts (19). All kinds of RNA m6A methylation regulators are involved in different physiological processes, while many remain unknown.

In this article, we introduce the novel RNA modification and its regulatory function for RNA. We summarize the main regulators of RNA m6A methylation and describe their function and regulatory mechanism toward mRNA. The possible target gene by which RNA m6A methylation regulators affect lipid metabolism is claimed. Finally, we reviewed the RNA m6A methylation regulators on the NAFLD, diabetes, and cardiovascular diseases and its regulating pathway to provide some reference to the clinical prevention, diagnosis, and therapy research in lipid metabolism-related diseases.

## Epigenetic regulatory mechanisms of RNA m6A methylation

M6A methylation is a newly discovered epigenetic regulatory mechanism in recent years. Among the more than 170 RNA modifications (20), m6A modification accounts for a large proportion in eukaryocyte (21). It is a methylation substitution reaction that takes place on the sixth nitrogen atom of the RNA molecule adenosine, which is observed enriching in 3’UTR and consensus motif RRACH in coding region (22, 23).

M6A methylation is essential in determining the fate of RNA, showing a regulatory function in multiple mRNA biological processes. Firstly, it can regulate the stability of mRNA. Facts that mRNA with lower m6A methylation level had longer half-life was first revealed in 1978 (24). The m6A reader YTHDF2 can recognize methylation sites in the coding region of mRNA and destabilized mRNA (25, 26) while, the newly identified reader Insulin-like growth factor-binding proteins (IGFBP) recognized m6A in 3‘UTR inversely make the mRNA more stable (27, 28). The opposite regulatory effect may account for the different recognizing mRNA sites. Secondly, m6A facilitates the initiation of the translation process of mRNA. After reading the m6A methylation site, the m6A reader like YTHFD1/3 can recruit eIF3 to connect to mRNA. In addition, m6A at 5‘UTR can directly connect to eIF3 to enhance mRNA translation (29–31). Furthermore, it also regulates mRNA splicing, processing, and nuclear export (32, 33). Recent research also shows it to to exist in lncRNA, microRNA, and non-coding RNA (32, 34, 35), considered a widespread RNA modification.

In RNA molecules, methylation levels are regulated by a series of enzymes reversibly and dynamically, which can be identified as “Writer”, “Eraser”, and “Reader” and all specifically interact with the m6A methylation site as follow.

### The writer of m6A can catalyze mRNA methylation

METTL3 is a high molecular weight subcomplex whose component is still not fully understood, and METTL14 is its homologues (21, 36). WTAP is the regulatory subunit of methyltransferase by which METTL3 and METTL14 anchor to mRNA to methylate subsequent target adenosine residues. WTAP recruits METT3 and METT14, enabling the METTL3- METT14 complex to perform m6A methyltransferase activity, affecting m6A methylation, and thus RNA shearing (37). Junho Choe’s team found that METTL3-elF3h interacts with each other to mediate mRNA cycling and translation through the association between the elF3h subunit at the mRNA 5‘end and METTL3 binding to the specific site near the translation termination codon. METTL3-elF3h mediates mRNA cyclization. Thus, efficient translation of target mRNA was promoted (38).

### The eraser of m6A can remove m6A from RNA

FTO was the first eraser to be identified, in 2011 (21). Since its discovery, much research on its regulation in enormous physiological and pathological processes has been carried out. FTO in humans is an approximate 400 kb gene, containing 8 introns and 9 exons, located on 16q12.2 (39). FTO can remove the m6A methylation from multiple mRNAs through an α-Ketoglutarate (α-KG) and Fe (II)-dependent manner (40). Its modification process is claimed in detail in previous research. In brief, initially, FTO oxidizes m6A methylation to the intermediate N6-hydroxymethyl adenosine (hm6A). In the second step, FTO oxidizes metastable hm6A in the same way as m6A, forming further oxidized production N6 -formyladenosine (f6A) (41). As a result, hm6A and f6a spontaneously break down to adenine and the m6A methylation in RNA is removed (41, 42).

ALKBH5 is another eraser identified later which demethylates the RNA efficiently (43). Research has shown its regulator function in many regulator pathways by mRNA methylation. However, the underlying mechanism remains mysterious.

### The reader of m6A can capture mRNA methylation

YTH domain is a module recognizing the methylation of m6A dependently, consisting of YTHDC1, YTHDC2, YTHDF1, YTHDF2, and YTHDF3 (25). The stability of m6A methylation modified mRNA is regulated by YTHDF2 in the way of recognizing m6A methylation and reducing the stability of the target transcript. In addition, another m6A reading protein, YTHDF1, was found to interact with the translation machinery of the related genes and promote protein synthesis. The m6A mRNA modification enforces rapid response of gene expression and controlled protein production, improved translation efficiency through YTHDF1-mediated translation, and controls target transcripts’ lifetime through YTHDF2-mediated degradation (29).

## RNA m6A methylation regulates the lipid metabolism

Lipid, which mainly consists of triglycerides, cholesterol, phospholipids, and glycolipid, is involved in body energy metabolism and is the component of the cell membrane. It is also the precursor of various molecules that play important biological roles. Thus, lipid metabolism, such as digestion, absorption, synthesis, and decomposition is essential for the maintenance of cellular homeostasis (44, 45).

**Figure 1:**
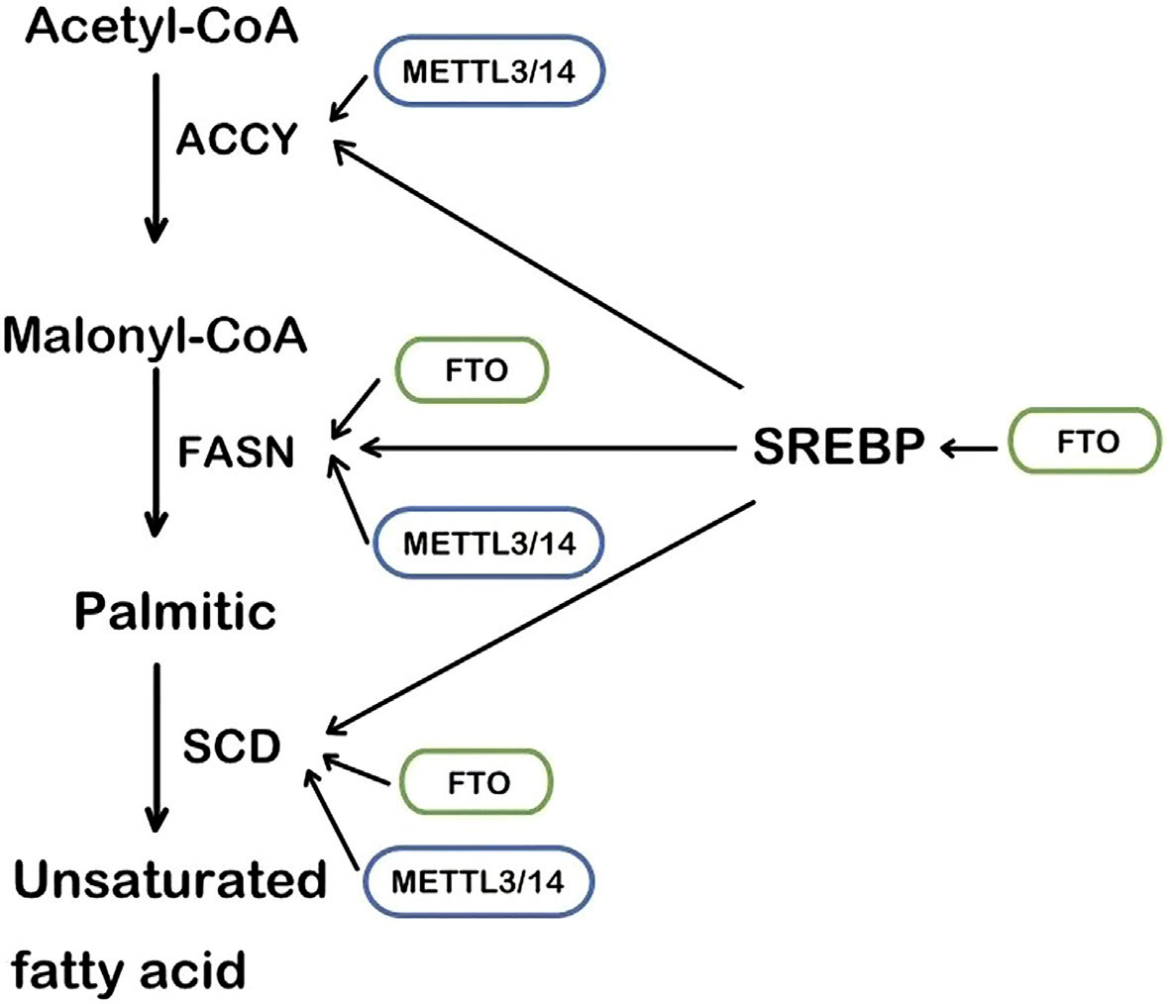
The main steps of lipogenesis and the regulation of FTO and METTL3/14. SREBP, sterol regulatory element-binding protein; FASN, fatty acid synthase; ACCY, acetyl-CoA carboxylase; SCD, stearoyl-CoA desaturase.

The RNA m6A methylase METTL3 and METTL4 are also involved in the regulation of lipid accumulation in cells. METTL3-mediated m6A methylation makes the metabolism-related gene’s mRNA more unstable, leading to metabolic disorders and lipid accumulation in the liver (46). Likewise, in cardiac cells, METTL3 deficiency decreases the RNA m6A methylation and the triglyceride deposition (47). Fatty acid synthase (FASN), acetyl-CoA carboxylase (ACCY), and stearoyl-CoA desaturase 1 (SCD1) are the regulator targets, as recently reported (**Figure 1**). Mechanistically, METTL3/METTL14 complex induces the increase of mRNA to accelerate the production of lipid (48, 49). Consistently, METTL3 and the recognizing and binding protein YTHDF2 increase the m6A methylation level of peroxisome proliferator-activated receptorα(PPARα) and its expression, impacting the downstream lipid accumulation (50). Inflammation is also involved in the lipid accumulation procedure. METTL3 deficiency induces a lower level of m6A methylation of TNF receptor-associated factor 6 (TRAF6) and therefore the transcripts are entrapped in the nucleus, leading to the downstream mitogen-activated protein kinase (MAPK) and nuclear factor κ-B (NF-κB) to be suppressed. In consequence, inflammation and the absorption of long-chain fatty acids (LCFA) are reduced (51).

Once introduced in 1974 (52), RNA m6A methylation modification was found to affect diverse physiological and pathological progressions in cardiomyocytes (53), hepatocyte (54), axoneuron (11), and so on. Its regulation function in the lipid metabolism is revealed over decades. In general, its regulation function depends on the enhancement or reduction of the m6A level and recognition of the m6A site by various regulatory enzymes. But its interaction with genes related to lipid synthesis and decomposition is complicated and remains to be elucidated by research.

The first identified RNA m6A demethylase, FTO, is strongly connected with lipid accumulation in multiple cells and tissues. In the obesity group, high FTO level is positively correlated with Body Mass Index (BMI) and body fat (55, 56). *In vitro*, it promotes intracellular lipid accumulation by RNA demethylation while FTO knockdown did not (57, 58).

As an enzyme that demethylates m6A (59), FTO regulates m6A methylation levels of multiple RNA in lipid anabolism and catabolism. The process of lipid synthesis can be improved by FTO-mediated RNA demethylation. In the 3’UTR region of multiple lipogenic genes’ mRNA such as SCD, PPARγ, and sterol regulatory element-bindin protein-1 (SREBP1), which are all involved in the triacylglycerol and Cholesterol Synthesis. (**Figure 1**) FTO decreases their level of m6A methylation to improve the stability of mRNA (60, 61). In the hepatocyte, the m6A methylation level in FASN mRNA is enhanced and lipogenesis is inhibited by the FTO knockdown and YTHDF2 recognition (62). Angiopoietin-like protein 4 (ANGPTL4) is also the key target of triglycerides synthesis and hydrolysis intracellularly and extracellularly. It inhibits lipoprotein lipase(LPL), leading to inhibiting extracellular lipolysis (63). FTO decreases the level of the translation of ANGPTL4, hence hydrolysis of extracellular triglycerides is promoted. The fatty acid is transported into adipocytes, inducing lipid accumulation (39, 63). Conversely, ANGPTL4 promotes intracellular lipolysis (64). Evidence has shown knockout of FTO affects intracellular ANGPTL4 level and intracellular lipolysis (65). The different results may account for the different mRNA sites where the m6A methylation is located. It is an interesting issue to explore.

Nevertheless, the role played by FTO in lipolysis remains disputed. FTO decreases the expression of interleukin 6 (IL-6) mRNA in adipose tissues (66) and consequently inhibits the lipolysis genes (67). In addition, FTO reduces lipolysis and fatty acid oxidation by reducing the adipose triglyceride lipase (ATGL), hormone-sensitive lipase (LIPE), and carnitine palmitoyltransferase 1 (CPT1) mRNA expression (68).

Interestingly, another FTO regulator pathway revealed that the promotion of FTO downregulated the obesity-related gene iroquois homeobox protein 3 (IRX3) level in the hypothalamus and macrophage. So, lipolysis was inhibited through affecting whole body modulated energy expenditure and metabolic inflammation (69, 70). However, it should be noted that the interaction of FTO and IRX3 is not the traditional m6A methylation modification, but the noncoding regions of FTO serve as a long-range regulatory element to influence the expression of IRX3 (71).

Furthermore, FTO-mediated RNA m6A methylation shows a close correlation with cellular triglyceride (TG) uptaking that is regulated by adenosine 5’-monophosphate-activated protein kinase (AMPK) (72, 73). AMPK suppresses the expression of FTO to upregulate the m6A level of Parkin2 mRNA and promote its decay. Then CD36 was translocated to the membrane and LCFA uptaking of cells is increased (74, 75).

**Figure 2:**
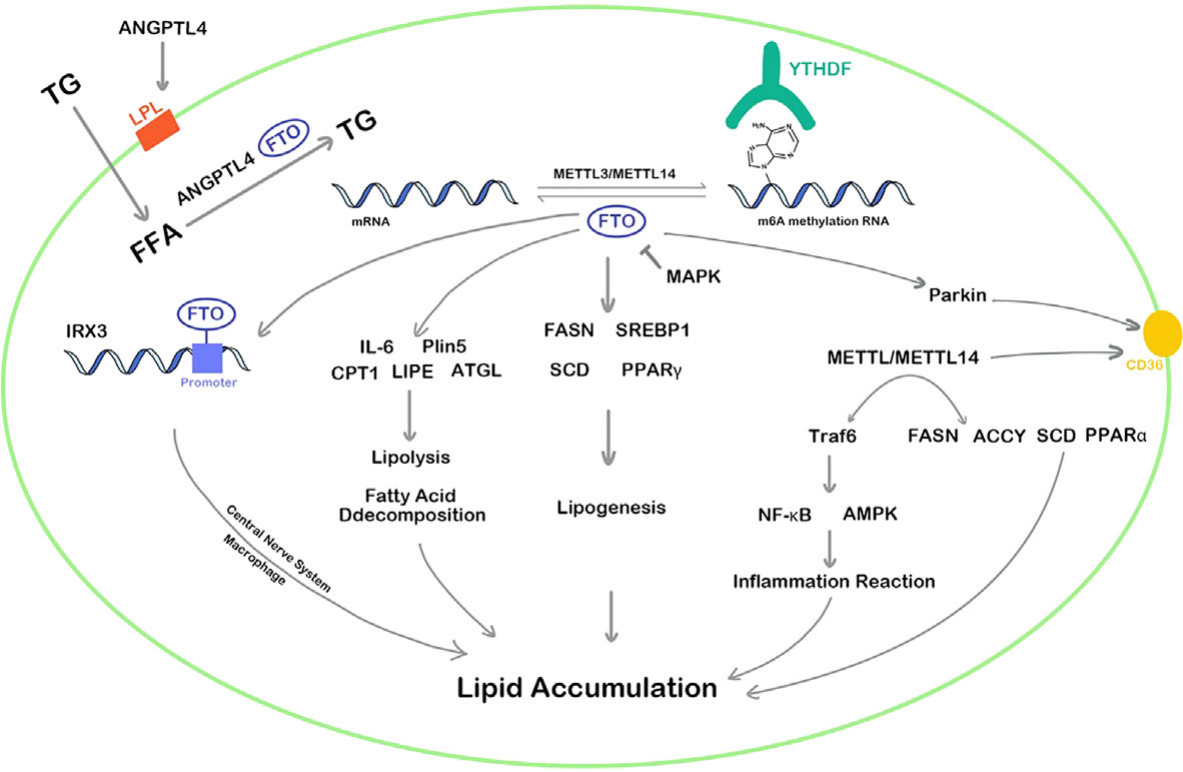
RNA m6A regulators influence lipid cellar accumulation in various ways. TG, triglyceride; ANGPTL4, angiopoietin-like protein 4; IRX3, iroquois homeobox protein 3; MAPK, mitogen-activated protein kinase; FFA, free fatty acid; IL-6, interleukin 6; Plin5, perilipin5; CPT1, carnitine palmitoyltransferase 1; LIPE, hormone-sensitive lipase; ATGL, adipose triglyceride lipase; PPAR, peroxisome proliferator-activated receptor; FASN, fatty acid synthase; LPL, lipoprotein lipase; SREBP1, sterol regulatory element-bindin protein-1; SCD, stearoyl-CoA desaturase; Traf6, TNF receptor associated factor 6; ACCY, acetyl-CoA carboxylase; NF-κb, nuclear factor kappa-B; AMPK, adenosine 5’-monophosphate-activated protein kinase.

In summary, both FTO and METTL3 play vital regulatory roles in lipid metabolism and can promote the accumulation of lipids in various cells, affecting the decomposition and synthesis of lipids. The regulation pathways of FTO and METTL3/METTL14 are complex and diverse, which can methylate or demethylate the RNA m6A of targets in multiple pathways such as inflammation, energy homeostasis, nerve-related lipid regulation, lipid metabolism balance, resulting in corresponding high or low gene expression (**Figure 2**). In addition, YTHDF protein plays an epigenetic role in recognizing m6A methylation sites of RNA and regulates the translation. Although the area of RNA m6A methylation is a popular spot in recent years, a convincing and authoritative theory is urgently needed. The function of RNA m6A methylation in many genes remains controversial and the deeply regulation process requires further investigation.

## m6A methylation and lipid-related metabolic diseases

**Figure 3:**
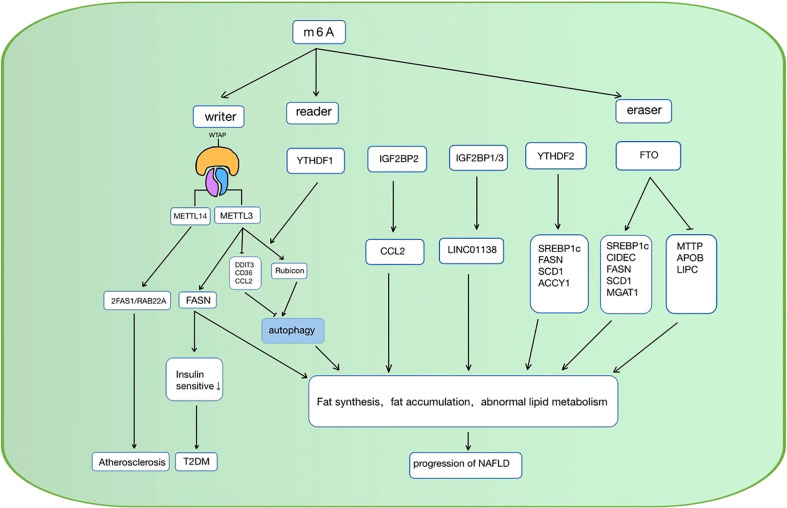
RNA m6A regulators are involved in the regulation of lipid metabolic diseases in various ways. NAFLD, non-alcoholic fatty liver disease; CCL, C-C motif chemokine ligand 2; SREBP1c, sterol regulatory element-bindin protein-1; FASN, Fatty acid synthase; SCD1, stearoyl-CoA desaturase; ACCY1, acetyl-CoA carboxylase, CIDEC, cell death-inducing DFF45-like effector C; MAGAT, monoacylglycerol acyltransferases; LIPC, hepatic lipase; APOB, apolipoprotein; ZFAS1/RAB22a, zinc finger antisense 1/ras-related protein rab-22a; DDIT3, DNA damage-inducible transcript 4; T2DM, diabetes mellitus type 2; METTL, methyltransferase-like 3; YTHFD, YT521-B homology domain family; FTO, fat mass and obesity-associated protein; IGFBP, Insulin-like growth factor-binding proteins; MTTP, microsomal triglyceride transfer protein.

When the cellular lipid metabolism is disordered, excessive lipid accumulation or lipid accumulation in ectopic tissues due to the imbalance of lipid uptake, decomposition, and synthesis in the cell, can result in a series of intracellular pathophysiological reactions. Inflammation (76), oxidative stress (77), chromatin histone modification (78), etc. caused by lipid accumulation can lead to cellular dysfunction, apoptosis, and even death. As mentioned above, RNA m6A methylation is involved in multiple pathways in lipid metabolism, and it also shows a vital function in the occurrence and development of lipid metabolic diseases (**Table 1**). Over the past decades, studies have investigated some possible targets for the diagnosis, physiopathology process, and therapy of metabolic diseases such as NAFLD, diabetes, hyperlipidemia, and atherosclerosis (**Figure 3**).

**Table 1:** Multiple functions of RNA m6A methylation regulator in lipid metabolic disease.

Regulator	Disease	Influence towards disease	Target	Function	Year	Ref.
METTL3	NAFLD	NEGATIVE	DDIT3	Loss of METTL3 results in increasing in DDIT	2021	(79)
METTL3	NAFLD	NEGATIVE	Rubicon axis	METTL3 and its partner YTHDF1 promote the stability of Rubicon mRNA	2021	(80)
METTL3	NAFLD/NASH	POSITIVE	CD36,CCL2	METTL3 inhibits the expression of CD36 and CCL2	2015	(81)
FTO	NAFLD	NEGATIVE	SREBP1c, CIDEC	Knockdown of FTO down-regulates the expression of SREBP1c and CIDEC	2018	(82)
FTO	NAFLD	NEGATIVE	FASN, SCD, MGAT1, MTTP, APOB, LIPC	FTO overexpression in HepG2 cells positively regulate FASN, SCD1, MAGT1 while negatively regulate MTTP, APOB, LIPC	2018	(57)
IGF2BP2	NAFLD	NEGATIVE	CCL2	Overexpression of p62/IMP2-2/IGF2BP2-2 elevated CCL2 expression levels	2014	(83)
IGF2BP1/IGF2BP3	NAFLD/HCC	NEGATIVE	LINC01138	IGF2BP1/IGF2BP3 stabilized LINC01138 transcript	2018	(84)
METTL3	T2DM	NEGATIVE	FASN	METTL3 silencing could decrease the m6A mRNA levels of FASN	2019	(48)
METTL3	HCC	NEGATIVE	SOCS	YTHDF2 cooperates with METTL3 depressing the level of SOCS	2018	(85)
YTHDC2	NAFLD	CRITICAL	SREBP1c, FASN, SCD1, and ACCY1	YTHDC2 decreases the stability of mRNA of SREBP1c, FASN, SCD1, and ACCY1 and inhibit gene expression	2020	(86)
FTO	HYPERLIPIDAEMIA	NEGATIVE	–	the secretion of inflammatory factors IL-1βand the expression of FTO was high in dyslipidemia induced by LPS	2016	(87)
METTL14	ATHEROSCLEROSIS	NEGATIVE	ZFAS1/RAB22a	METTL14 mediated m6A modification to LncRNA ZFAS1/RAB22a	2020	(88)

### m6A methylation and the lipid metabolism in NAFLD

The liver is one of the most significant organs in fatty acid synthesis and decomposition. Recent studies have revealed that RNA m6A methylation happened in hepatocyte matters in lipid metabolism disorder. Patients who suffered from NAFLD were detected to have a higher level of FTO mRNA in the liver (46). Similar results were observed in several studies (89–91), which have been widely acknowledged by researchers.

Thus, exploring the further mechanism is imperative. The “writer” METTL3 is also considered to be related to liver lipid accumulation (50, 92). Forkhead box O1 (FOXO1), Enoyl-CoA Hydratase And 3-Hydroxyacyl CoA Dehydrogenase (EHHADH), PPARα, FASN, and Sirtuin 1 (SIRT1) were the regulator targets that had been reported (93). Furthermore, in the recent 2 years of research, some other regulation targets have been put forward. METTL3, as the m6A writer, improves DNA damage-inducible transcript 4 (DDIT4) mRNA the methylation level, as a result, affects its stability. When METTL3 is knocked down, DDIT4 reduces the level of lipid accumulation and the activity of inflammation in hepatocytes of the NAFLD patients by the signaling pathway of the mechanistic target of rapamycin complex 1 (mTORC1) and NF-κB (79). Autophagy also plays a role in RNA m6A methylation of the NAFLD progression. METTL3 and its partner YTHDF1 inhibit the autophagic flux in hepatocytes and block the clearance of lipid droplets by the means of promoting the stability of Rubicon mRNA, which inhibited the autophagy process of autophagosome-lysosome fusion (80).

Conversely, METTL3 knockdown increased the free fatty acid uptake mediated by CD36, and the inflammation reaction induced by C-C motif chemokine ligand 2 (CCL2), as the result, lead to the progression from NAFLD to non-alcoholic steatohepatitis (NASH) (81). The regulation of METTL3 on NAFLD may be diverse.

FTO can affect the expression of FASN, SCD, Monoacylglycerol acyltransferase (MAGAT), SREBP1c, and cell death-inducing DFF45-like effector C (CIDEC) (82) to regulate the lipogenesis in hepatocytes. Meanwhile, FTO up/down-regulates the lipid transport protein of microsomal triglyceride transfer protein (MTTP), hepatic lipase (LIPC), apolipoprotein B (APOB) (57), inducing the process of lipid transport (93). As a result, excessive lipid deposition in hepatocytes results in hepatocyte steatosis.

Furthermore, the “reader” YTHDF2 is also involved in the regulation of TG homeostasis and lipogenesis in NAFLD, and SREBP1c, FASN, and SCD1, and ACCY1 is the gene related to the process (86). In the next section, IGF2BP2, a recently identified m6A reader, was also reported to be a promoter of NAFLD (83), which can promote the stability of mRNA (28), and IGF2BP1/IGF2BP3 was also reported to be associated with poor outcomes of liver cancer (84).

NAFLD is a complex metabolic disease and many pathological changes happened in liver tissue (94).The RNA m6A methylation regulator FTO, METTL3, and the recognition protein family YTHDF affect the progression of NAFLD to hepatocellular carcinoma (HCC) by the means of disorder lipid metabolism, oxidative stress (87), and autophagy (80), making it an important potential treatment target. Altering the RNA m6A methylation level of various proteins reduces the hepatic abnormal lipid accumulation, thereby further relieving the abnormal state of cells. This epigenetic regulation may significantly improve the development of NAFLD and even reverse hepatocyte degeneration.

### m6A methylation and lipid metabolism in diabetes

Diabetes is one of the highest prevalence diseases and over 400 million patients live with this disease worldwide. Its complication causes severe disease burden (95, 96). It has been revealed that multiple m6A methylations target pathways like Insulin-like growth factor 1-protein kinase B-pancreatic and duodenal homeobox 1(IGF1-AKT-PDX1) and genes like diacylglycerol acyltransferase 2 (DGAT2), glucose-6-phsophatase catalytic subunit (G6PC), and FOXO1, are involved in the glucose and insulin secretion regulation of pancreatic islet B cell (97, 98). Besides, lipid metabolism disorder related to m6A methylation also plays an important part in insulin resistance.

FASN, the key protein in lipid metabolism, has proved to be closely connected to insulin resistance by research that in adipose tissue, FASN expression was increased and insulin sensitivity was impaired (99). METTL3 also inhibits insulin sensitivity *via* the modification of FASN mRNA. Along with the overexpression or the METTL3 deficiency in high-fat diet (HFD) rats, the level of FASN mRNA and lipid content in the liver is higher or lower accordingly, and the insulin sensitivity is improved (46, 48). However, further studies are still needed to claim how exactly m6A interacts with insulin sensitivity.

### RNA m6A methylation and lipid metabolism in cardiovascular diseases

Cardiovascular disease is the leading cause of death worldwide, while hyperlipidemia is responsible for about one-third of all cardiovascular diseases (100, 101). The RNA m6A methylation involved lipid metabolism disorder and chronic inflammation reaction has been reported as a possible mechanism in the past few years. Research has revealed that hyperlipidemia level is highly connected with m6A-SNPs (102) and FTO-associated inflammatory factor IL-1β, IL-6, and LPS which induce hyperlipidemia may be the factors in the development of chronic heart disease (38, 87). Additionally, 6-phosphogluconate dehydrogenase (6PGD) is also considered a key point. YTHDF2 binds to 6PGD mRNA and promotes its translation, while 6PGD deficiency can lead to lower blood cholesterol making YTHDF2 a possible target for lowering blood cholesterol (103–105). Moreover, very recent research mentioned that the METTL14 mediating lncRNA zinc finger antisense 1/ras-related protein rab-22a (ZFAS1/RAB22a) m6A methylation modification is also a possible pathway to atherosclerosis (88).

## Conclusion and discussion

After decades of research, RNA m6A methylation remains a broad research space that structures functions and regulation mechanisms of many regulators remain critical and unknown (106). RNA m6A methylation is an important and novel regulatory manner in epigenetics. It has a regulatory role in adipogenicity differentiation and adipogenesis in adipose tissue (107, 108). In addition, it also exerts vital functions in lipid metabolism, which is interwoven with human health. A greater understanding of the regulatory mechanism of lipid metabolism also leads to advances in life science research.

In the process of RNA m6A methylation regulating the lipid metabolism, the m6A “writers”, “erasers”, and “readers” can add, remove, or recognize the RNA m6A methylation sites in mRNA and affect its translation, decay, splicing, and export, leading to thousands of biological processes (109). Inflammation is one of the parts, and it has proved closely connected with obesity and fatty acid absorption (110, 111). In the process of RNA m6A methylation regulating lipid metabolism, IL-6, CCL2, IRX3, TRAF6, and many inflammation factors-related proteins become the central regulatory targets. The lipid synthesis and decomposition genes such as FASN, SREBP, and CES2 (112) are also affected by mRNA m6A methylation and demethylation. Lipogenesis and lipolysis are directly regulated. In addition, some other cell signaling pathways are also involved. The regulation of RNA m6A methylation is a complex process that involves a variety of mechanisms in multiple cells. Research in this area still has much to be done.

Disorder of the global or partial lipid metabolism causes intractable chronic diseases. In the occurrence and development of NAFLD, abnormal lipid accumulation in hepatocytes is one of the major pathological changes, and METTL3, METTL14, FTO, and YTHDF mediated key gene mRNA m6A methylation are all related to it. Furthermore, lipid metabolism disorder is responsible for insulin resistance, hyperlipidemia, and atherosclerosis (113), in which RNA m6A methylation all plays a critical part, making it a great potential therapeutic target.

At the present stage, further research on m6A mRNA methylation in its effective metabolism is needed. Many studies remain controversial, and m6A mRNA methylation may affect the expression of mRNA or protein levels of different key positive or negative regulatory factors in different pathophysiological processes, so, likely, the “Writer”, “Eraser”, and “Reader” of the same RNA m6A methylation regulators may coregulate two pathophysiological processes with opposite effects. Moreover, future studies on the regulatory mechanism of m6A mRNA methylation on adipose metabolism should not be limited to METTL3, FTO, and YTHDF2, and other “Writer”, “Eraser”, and “Reader” of m6A mRNA methylation may also participate in the occurrence of lipid metabolism through different pathways while researches remain limited. In summary, RNA m6A methylation regulates many targets, including lipid synthesis, breakdown, as well as accumulation. Moreover, RNA m6A methylation has the therapeutic potential to be a target for metabolic diseases like obesity, NAFLD, and diabetes which will foster the treatment of them and related diseases better in humans in the future.

## Author contributions

YF conceived the study and designed the study protocol. YTW and YJW conducted the literature review and drafted the manuscript. TS and JG reviewed the manuscript for intellectual content, and XG made revisions as needed. All authors contributed to the article and approved the submitted version.

## Funding

This work was supported by the National Natural Science Foundation of China (Grant numbers 82070838 to FY, 82170831 to GX), National Tutorial System Training Project for Youth Key Talents in the Suzhou Health system(Grant numbers Qngg2021007 to FY), Project of medical application of nuclear technology in discipline construction(Grant numbers XKTJ-HRC2021007 to GX).

## Conflict of interest

The authors declare that the research was conducted in the absence of any commercial or financial relationships that could be construed as a potential conflict of interest.

## Publisher’s note

All claims expressed in this article are solely those of the authors and do not necessarily represent those of their affiliated organizations, or those of the publisher, the editors and the reviewers. Any product that may be evaluated in this article, or claim that may be made by its manufacturer, is not guaranteed or endorsed by the publisher.
